# Mature cystic teratoma of the temporal lobe: A rare tumor with an unusual location

**DOI:** 10.1002/ccr3.5340

**Published:** 2022-02-02

**Authors:** Yasoda Rijal, Om Bikram Shah, Suraj Shrestha, Bibek Man Shrestha, Shiva Lal Bhattarai, Sabita Bishowkarma, Gopal Sedhai

**Affiliations:** ^1^ Maharajgunj Medical Campus Institute of Medicine Kathmandu Nepal; ^2^ 92959 Department of Neurosurgery Tribhuvan University Teaching Hospital Kathmandu Nepal; ^3^ 92959 Department of Pathology Tribhuvan University Teaching Hospital Kathmandu Nepal

**Keywords:** germ cell tumor, seizure, temporal lobe, teratoma

## Abstract

Intracranial mature cystic teratoma is rare. The temporal lobe is a very unusual location and can pose a considerable diagnostic challenge. Proper histological diagnosis and long‐term follow‐up are imperative.

## Graphical abstract

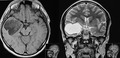

## 1 BACKGROUND

Central nervous system germ cell tumors (GCT) including intracranial teratoma are rare and comprise only 0.3%–0.6% of all primary intracranial tumors.^1^, ^2^ Most cases occur in a midline location usually in the pineal region, followed by the suprasellar/hypothalamic region.^2^ Non‐midline locations of these tumors are uncommon and can rarely occur in the basal ganglia, lateral ventricle, fourth ventricle, Sylvian fissure, cavernous sinus, and temporal lobe.^3^, ^4^, ^5^ A review of 44 cases of dermoid cysts found that tumors in the temporal lobe region accounted for 13.6% of all intracranial dermoid cysts, both immature and mature teratomas.^6^

The patient's clinical presentation is nonspecific, depending on the tumor size and location, with signs of raised intracranial pressure (ICP) as the most common.^4^ Seizure is an uncommon presentation of intracranial GCTs. Reports of mature cystic teratoma of the temporal lobe presenting as focal seizure with impaired awareness are rare.^7^, ^8^ Hoyer et al. had described a case of frontal and temporal lobe teratoma during the autopsy examination with probable generalized tonic‐clonic seizure as the cause of death attributing to the location of the tumor.^9^

Herein, we report the case of a 17‐year‐old male who presented with a focal seizure with impaired awareness and was subsequently diagnosed with primary mature cystic teratoma of the right temporal lobe.

## 2 CASE REPORT

A 17‐year‐old male presented with a history of abnormal movement of the lips and tongue for the past 6 years. Before the onset of an episode, he had an aura in the form of blurring of affect for a brief period, followed by multiple episodes of abnormal movements of the lips and tongue lasting for about 1 min which he was unaware of, with each episode lasting for approximately 4–5 s. He did not have generalized body movements, uprolling of the eyes, clenching of teeth, tongue bite, frothing from the mouth, and urinary/fecal incontinence during the attack. Following the episode, he experienced postictal confusion and headache for a few minutes. These seizure episodes had resulted in minor accidents, falls, and trauma. The frequency of these episodes gradually increased from once a month initially to 4–6 times a day in the past few months. There was no history of loss of consciousness and neurological deficits, limb weakness, visual diminution, fever, headache, weight loss, vomiting, or features suggestive of raised ICP.

On neurological examination, the Glasgow coma scale was 15/15 with regular reactive pupils and normal fundoscopy. All the cranial nerves were intact. Motor power was 5/5 (MRC grade) across all major joints with an intact sensory examination. All other systemic examinations were unremarkable.

**FIGURE 1:**
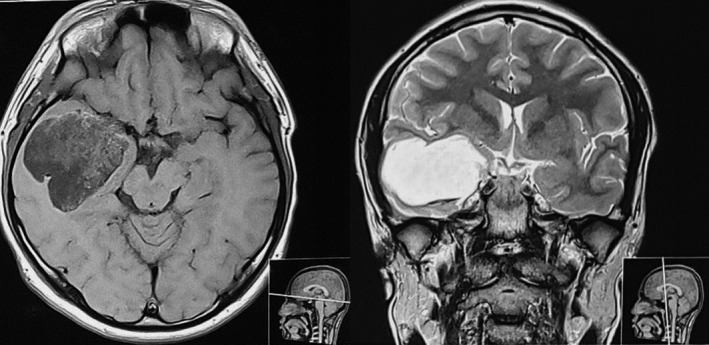
T1‐MRI (Axial section) showing intraaxial mass of variable intensity in the right temporal pole compressing adjacent ambient and Sylvian cistern (Left) and T2‐MRI (Coronal section) with bright signal intensity in the right temporal region (Right)

Magnetic Resonance Imaging (MRI) of the brain revealed approximately 5.8 cm × 5.5 cm × 5.2 cm sized well‐defined T1 heterogeneously low, T2 heterogeneously high‐signal intensity cystic mass with thin septations in the right temporal lobe with mild patchy restriction of diffusion in diffusion‐weighted imaging (DWI) image without any solid component in it. The lesion showed peripheral wall enhancement in post‐contrast images alongside mass effect causing effacement of adjacent sulci, right Sylvian fissure, and compression of the ipsilateral temporal horn of the lateral ventricle. (Figure 1) With a preoperative diagnosis of an epidermoid cyst with differentials of ganglioglioma and dysembryoplastic neuroepithelial tumor (DNET), the patient was planned for surgical removal of the tumor.

**FIGURE 2:**
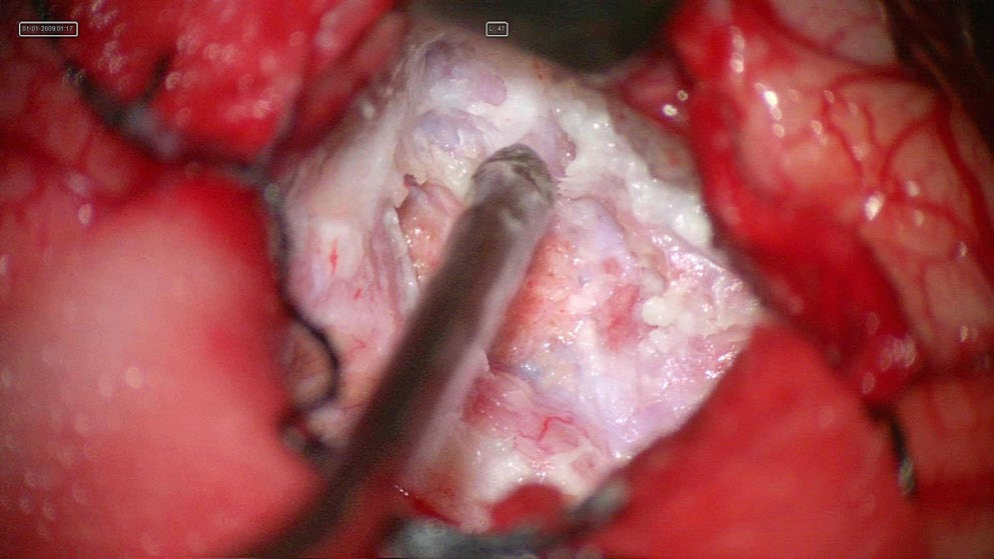
Intraoperative images showing tumor with easily suckable ivory white cheesy content

**FIGURE 3:**
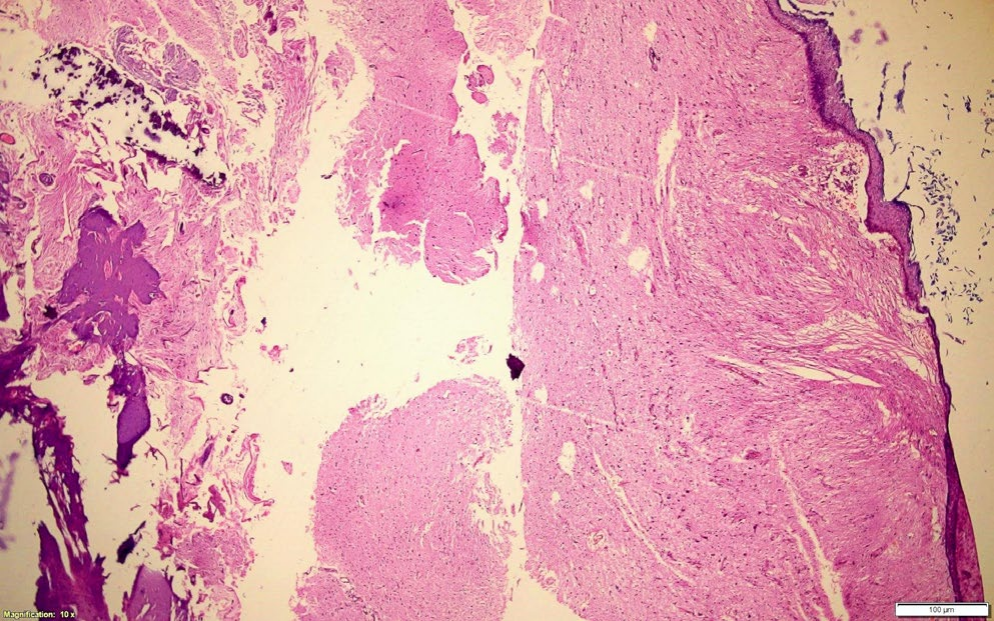
Section shows cyst lined by keratinized stratified squamous epithelium. The wall shows an area of calcification, mature glial tissues, and bony tissues (H&E, ×40)

He underwent right temporal craniotomy with gross total resection of the tumor. Intraoperatively, there was an easily suckable ivory white cheesy tumor involving the right temporal lobe with no vascularity and variable consistency at the base of the lesion. Intraoperative electrocorticography was done which revealed a positive signal at the base of the lesion, possibly the focus of the seizure. After the excision of the tumor, the electrocorticography was repeated and no signal was detected. (Figure 2) Histopathological examination of the excised mass showed cyst containing keratinous material, lined by keratinized stratified squamous epithelium with the wall showing calcification, mature glial tissues, bony tissues, multinucleated giant cells, and foreign body reaction confirmatory of mature cystic teratoma. (Figure 3) The post‐operative period was uneventful and the patient was discharged after 5 days of surgery. The patient is currently receiving sodium valproate 300 mg twice daily and is seizure‐free till 1 year of surgery and is under regular follow‐up.

## 3 DISCUSSION

Intracranial teratomas are rare tumors of the central nervous system(CNS). Depending on the degree of differentiation of the components' tissues, teratoma is classified as mature or immature; mature teratoma can be identified based on the presence of fully differentiated elements from all three germinal layers.^10^ According to the embryonic theory, the mis‐migration of the pluripotent germ cell is probably responsible for the growth of these tumors.^11^

CNS GCTs (Central nervous system germ cell tumors) have an overall male predominance and in a series of 254 teratomas by Tapper and Lack, most of the patients were aged 21 years or less.^11^, ^12^ Our patient was a 17‐year‐old male at the time of presentation.

As the tumor is often midline in the pineal region, obstructive hydrocephalus is the most common manifestation of CNS GCTs, and patients present with features of raised ICP. However, temporal/frontal lobe tumors are more frequently associated with seizures than other manifestations.^13^ Moreover, mature cystic teratoma/dermoid cysts have been reported to be associated with the new onset of seizure disorder. Potential sequelae of dermoid cysts which include rupture that can lead to inflammation or growth with expansion leading to compression of neural tissue can cause seizures.^14^ Generalized complex seizures are the most common type in reported cases among patients with intracranial dermoid tumors. Dermoid cysts in the temporal region rarely cause focal seizures with impaired awareness, previously known as complex partial seizures.^15^ Also, the dermoid cyst in the subdural space of the temporal region may manifest as generalized convulsions due to irritation of the brain surface caused by rupture and dissemination of the contents in the subarachnoid space.^16^ Our patient presented with an unusual focal seizure comprising of abnormal movements of the lips and tongue with impaired awareness without features of generalized convulsions.

MRI is considered the radiologic study of choice in the diagnosis and staging of the brain tumors, and the lesions of GCTs are hypointense on T1‐weighted hyperintense on T2‐weighted images, and non‐enhancing in contrast unless it has a cystic component. The presence of fatty tissue or multilocularity and calcifications is a characteristic predominant feature of teratoma.^17^, ^18^ Though dermoid cysts can be differentiated from the epidermoid cyst owing to its fat signal on MRI and epidermoid resembles CSF using Fluid‐Attenuated Inversion Recovery (FLAIR) sequences and DWI, dermoid cysts can often resemble epidermoids due to their bright signal on DWI.^19^, ^20^, ^21^ In our case, MRI finding of bright signal on DWI and absence of fatty tissue and calcification led to the preoperative diagnosis of an epidermoid cyst. Moreover, the intraoperative finding of ivory white cheesy consistency was also suggestive of intracranial epidermoid which after histopathologic examination turned out to be mature cystic teratoma. There were some areas of calcification noted only on HPE and were missed during surgical removal of the tumor.

Histopathological examination of mature teratoma shows a well‐differentiated tissue with low mitotic activity, while immature teratoma shows hypercellular embryonic mesenchyme or primitive neuroectodermal elements mimicking fetal tissue that can be mixed with mature tissue elements with a rare incidence of malignant transformation.^22^, ^23^ There were no malignant components seen in our specimen. However, it is pivotal to determine the true histopathological diagnosis with extensive sampling, as even the presence of a minor part of the immature tissue can change the treatment option.^24^

The treatment strategy of teratoma is still controversial. Gross total excision should be done whenever possible and the surgical approach depends on the location of the teratoma. Total excision of the tumor is the treatment of choice for mature teratomas as they are benign and usually radioresistant.^25^ In our case, temporal craniotomy with gross total excision of the tumor was done. Radiotherapy or chemotherapy plays a role for tumors showing immature or malignant components.^26^ The current general policy is that if total removal of a mature teratoma has been performed, adjuvant therapy is not strictly necessary in principle. However, misdiagnosis of teratoma histological subtypes leads to inadequate therapy, so careful histological analysis of the entire specimen is very important. Adjuvant therapy may be necessary after partial removal of a mature teratoma.^25^, ^27^

The 10‐year survival is as high as 93% in one large series, which signifies an excellent prognosis of intracranial mature teratomas.^28^ While there is uneventful recovery for most of the patients, malignant teratomas or new teratomas may arise intra‐ or extracranially, so they should be followed up for a longer period.^22^

## 4 CONCLUSION

Intracranial germ cell tumors are rare with the temporal lobe being an unusual location. Moreover, mature teratoma presenting as a focal seizure is not common. Nonetheless, mature cystic teratoma should be considered as a differential diagnosis of intracranial tumors presenting with seizures in young patients.

## CONFLICT OF INTERESTS

None to declare.

## AUTHOR CONTRIBUTION

GS and OS: did study concept, data collection, and surgical therapy for the patient. YR and SS: did writing – original draft preparation. BMS, SLB, and SB: did editing and writing. GS and OS: are senior authors and manuscript reviewers. All the authors read and approved the manuscript.

## CONSENT

Written informed consent was obtained from the patient's father for publication of this case report and accompanying images. A copy of the written consent is available for review by the Editor‐in‐Chief of this journal on request.

## Data Availability

All the necessary data and materials are within the manuscript.
